# Chinese Herbal Medicine Therapy Reduces the Risks of Overall and Anemia-Related Mortalities in Patients With Aplastic Anemia: A Nationwide Retrospective Study in Taiwan

**DOI:** 10.3389/fphar.2021.730776

**Published:** 2021-10-08

**Authors:** Mu-Lin Chiu, Yu-Lung Hsu, Chao-Jung Chen, Te-Mao Li, Jian-Shiun Chiou, Fuu-Jen Tsai, Ting-Hsu Lin, Chiu-Chu Liao, Shao-Mei Huang, Chen-Hsing Chou, Wen-Miin Liang, Ying-Ju Lin

**Affiliations:** ^1^ School of Chinese Medicine, China Medical University, Taichung, Taiwan; ^2^ Proteomics Core Laboratory, Department of Medical Research, Genetic Center, China Medical University Hospital, Taichung, Taiwan; ^3^ China Medical University Children’s Hospital, China Medical University, Taichung, Taiwan; ^4^ Graduate Institute of Integrated Medicine, China Medical University, Taichung, Taiwan; ^5^ Department of Health Services Administration, China Medical University, Taichung, Taiwan; ^6^ Department of Biotechnology and Bioinformatics, Asia University, Taichung, Taiwan

**Keywords:** aplastic anemia, overall mortality, anemia-related mortality, Chinese herbal medicine, network analysis

## Abstract

Aplastic Anemia (AA) is a rare but fatal hematologic disease that may occur at any age and especially higher in Asia. We investigated whether Chinese herbal medicine (CHM) is beneficial to AA patients as a complementary therapy using a nationwide population-based database in Taiwan between 2000–2016. Patient survival was estimated by Kaplan‒Meier survival analyses and Cox proportional-hazard model. CHM-users presented lower risks of overall and anemia-related mortalities when compared to non-users. The risk of overall mortality for CHM-users in AA patients was 0.70-fold [adjusted hazard ratio (aHR): 0.70, 95% confidence interval (CI): 0.66-0.74, *p* < 0.001). The risk of anemia-related mortality was lower in CHM-users when compared to non-users (aHR: 0.46, 95% CI: 0.32-0.67, *p* < 0.001). The association rule analysis revealed that CHM pairs were Ban-Zhi-Lian (BZL; *Scutellaria barbata* D. Don)→Bai-Hua-She-She-Cao (BHSSC; *Oldenlandia diffusa* (Willd.) Roxb.), followed by Dang-Gui (DG; *Angelica sinensis* (Oliv.) Diels)→Huang-Qi (HQi; *Astragalus membranaceus* (Fisch.) Bunge), and Xian-He-Cao (XHC; *Agrimonia pilosa f. borealis* (Kitag.) Chu)→Gui-Pi-Tang (GPT). Network analysis showed that BZL, BHSSC, DG, HQi, XHC, GPT, and Dan-Shen (DanS; *Salvia miltiorrhiza var. charbonnelii* (H.Lév.) C.Y.Wu) were commonly used CHMs for AA patients. Therefore, further studies for these commonly prescribed herbs are needed in functional investigations in hematopoiesis-stimulating effect and large-scale randomized controlled trials (RCT) in bone marrow failure related diseases.

## Introduction

Aplastic anemia (AA) is a rare but fatal hematologic and auto-immune disease (Clucas et al., 2019; Furlong and Carter, 2020), which mainly occurs in individuals from Asian countries (Vaht et al., 2017; Akram et al., 2019; Li et al., 2019). The incidence of AA is below 2.5 cases/million/year in Europe and America; however, higher incidence of AA is observed with 7.4 cases/million/year in China, 4.8 cases/million/year in Malaysia, and 5.67 cases/million/year in Taiwan (Vaht et al., 2017; Li et al., 2019). AA may occur at any age and is characterized with bone marrow failure syndrome and pancytopenia in peripheral blood (Peslak et al., 2017). It is known that immune-mediated destruction of hematopoiesis is the major pathogenesis of AA (Medinger et al., 2018). In AA patients, dysregulated and self-reacted cytotoxic T cells secrete pro-inflammatory cytokines, target hematopoietic stem and progenitor cells, induce cell death, and result in hematopoietic failure (Socie et al., 1993; Nakao et al., 2005; Zeng and Katsanis, 2015; Medinger et al., 2018; Ding et al., 2019; Liu et al., 2020).

The major AA treatment includes anti-thymocyte globulin (ATG)-based immunosuppressive therapy (IST) and hematopoietic stem cell transplantation (HSCT) (Speck et al., 1981; Bacigalupo et al., 2000). Other treatment includes glucocorticoid usage, blood transfusion therapy, hematopoietic growth factor therapy, chemotherapy, iron chelation therapy, and androgen therapy etc (Gale et al., 1981; Gurion et al., 2009). With these treatments in patients with AA, the 5-years survival rate approaches 60–80% (Vaht et al., 2017; Li et al., 2019). However, IST belongs to lymphocytotoxic agents, and some of these agents have cause complications, such as anaphylaxis fever, chest pain, diarrhea, infections, and subsequent malignant conditions (Socie et al., 1993; Passweg and Aljurf, 2013; Fu et al., 2017; van der Hem et al., 2017). For HSCT, it is very hard to find a matched donor, making this treatment less favorable when compared with IST (Zhu et al., 2016). Therefore, modulating anti-inflammatory, anti-complement, anti-cancer, anti-oxidant, anti-microbial, immune-modulating as well as hematopoiesis-stimulating based therapeutic strategies may be beneficial for AA patients (Socie et al., 1993; Nakao et al., 2005; Fu et al., 2017; van der Hem et al., 2017; Chatterjee and Law, 2018; Ding et al., 2019; Liu et al., 2020).

There is a need for seeking alternative therapies for the possible combination with the current conventional therapies. Studies in Chinese herbal medicines (CHMs) and related natural compounds show as effective, safe, less toxic and few side-effects in bone marrow failure related diseases (Gao and Chong, 2012; Zhu N. et al., 2018; Sun et al., 2018; Liu WB. et al., 2019). Furthermore, In Taiwan, CHMs have been widely used in many diseases (Lin et al., 2015; Li et al., 2018; Lee et al., 2019; Tsai et al., 2019; Wang CY. et al., 2020; Wu et al., 2020; Ho et al., 2021). Taiwanese AA patients may also choose CHM as their integrative, alternative, and complementary therapy to reduce complications from conventional therapies and to improve the overall survival rate.

In this study, we therefore used a nationwide population-based database in Taiwan to perform a retrospective cohort study and evaluate the CHM effect on overall and anemia-related mortalities for patients with AA. The CHM prescription pattern was also investigated from those with lower risks of overall and anemia-related mortalities.

## Materials and Methods

### Database Source

We used a longitudinal medical claims data, National Health Insurance Research Database (NHIRD) of Taiwan, to investigate the risks of overall and anemia-related mortalities between CHM and non-CHM users in AA patients during the period between January 1, 2000 and December 31, 2016. In total, 28,867,331 beneficiaries were included in this database. The diseases were identified by the International Classification of Disease, Ninth Revision, Clinical Modification (ICD-9-CM) codes. The informed consent was not demanded because all of the personal data were de-identified. The Institutional Review Board (IRB) issued the ethical approval to this study (CMUH107-REC3-074 (CR1)) at China Medical University Hospital.

### Study Subjects

**FIGURE 1:**
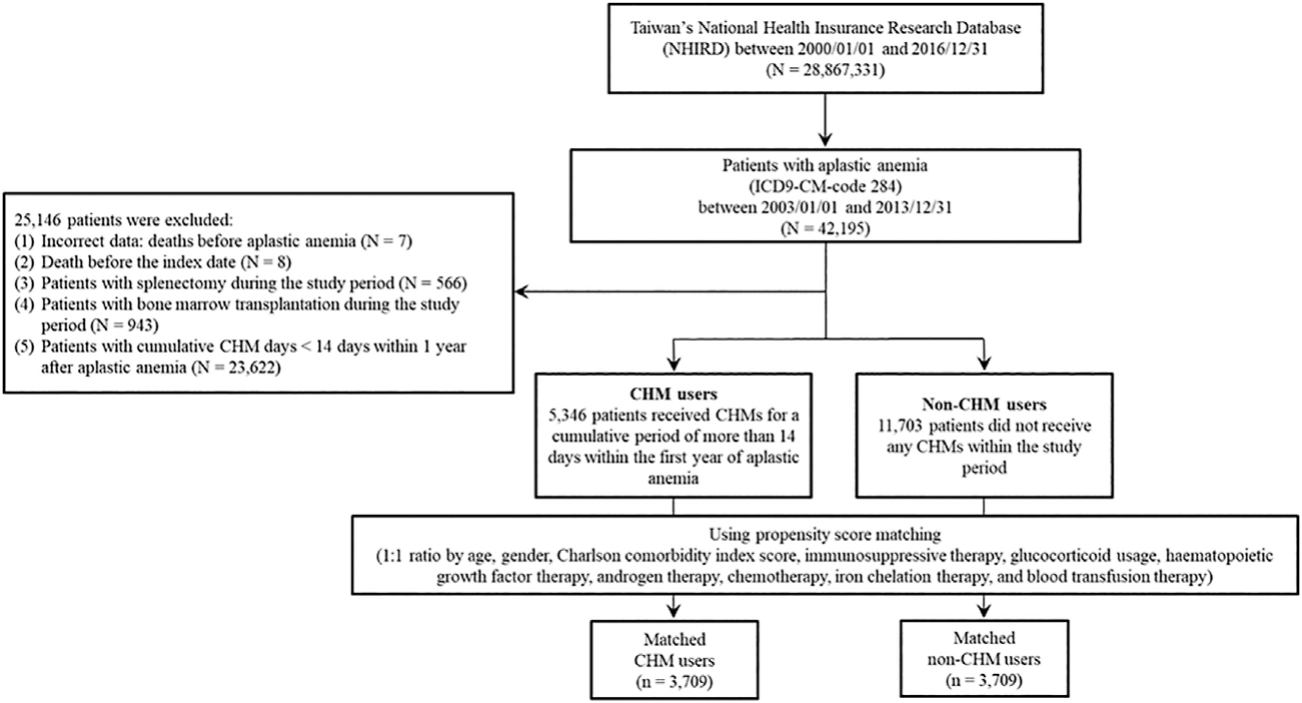
Flowchart of aplastic anemia patient enrollment.

**FIGURE 2:**
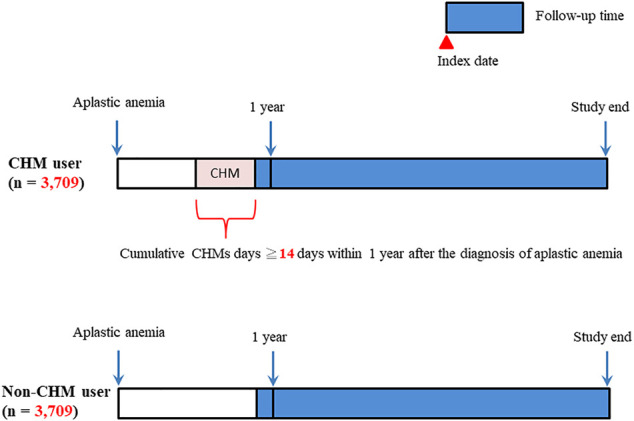
Diagram of follow-up time for AA patients. Abbreviations: AA, aplastic anemia.

In this study, there were 42,195 patients with aplastic anemia (AA) (ICD-9-CM-code: 284) identified during the period between January 1, 2003 and December 31, 2013 (Figure 1). We excluded 25,146 patients with AA. These excluded AA patients were with incorrect data (*N* = 7), death before the index date (*N* = 8), splenectomy during the study period (N = 566), bone marrow transplantation during the study period (*N* = 943), and cumulative CHM days <14 days within 1 year after AA diagnosis (*N* = 23,622). During the follow-up period, there were 5,346 CHM-users and 11,703 non-CHM users among AA patients. To eliminate potential bias caused by confounders, the CHM users and non-users were matched by age, gender, Charlson comorbidity index (CCI) score, immunosuppressive therapy, glucocorticoid usage, hematopoietic growth factor therapy, androgen therapy, chemotherapy, iron chelation therapy, and blood transfusion therapy using the propensity score matching method at 1:1 ratio. After matching, 3,709 CHM-user and 3,709 non-CHM-user matched pairs were identified (Figure 1 and Table 1). The date with a completed accumulation of 14 CHM days within 1 year after AA diagnosis was designated as the index date (Figure 2). Those who continued to use CHMs after the index date were designated as the CHM-users. The study endpoint was the end of 2016, death, or withdrawal from the NHIRD.

**TABLE 1:** Basic characteristics of patients with aplastic anemia in Taiwan.

Characteristics	Total subjects		*p*-value	Matched subjects		*p*-value
	CHM users (N = 5,346)	Non-CHM users (N = 11,703)		CHM users (N = 3,709)	Non-CHM users (N = 3,709)	
	N (%)	N (%)		N (%)	N (%)	
Age (years old)	—	—	—	—	—	—
0≦Age<18	340 (6.36%)	985 (8.42%)	***<0.001***	314 (8.47%)	304 (8.20%)	0.699
18≦Age<40	848 (15.87%)	844 (7.21%)	—	436 (11.76%)	420 (11.32%)	—
40≦Age<65	2,463 (46.08%)	3,994 (34.13%)	—	1,660 (44.76%)	1,639 (44.19%)	—
65≦Age	1,694 (31.69%)	5,878 (50.24%)	—	1,299 (35.02%)	1,346 (36.29%)	—
Gender	—	—	***<0.001***	—	—	0.674
Male	2090 (39.31%)	7,275 (62.68%)	—	1,626 (43.84%)	1,644 (44.32%)	
Female	3,227 (60.69%)	4,332 (37.32%)	—	2083 (56.16%)	2065 (55.68%)	
Charlson comorbidity index score (CCI score; Mean ± SD)	4.17 ± 3.52	4.93 ± 3.51	***<0.001***	4.29 ± 3.46	4.25 ± 3.34	0.554
Charlson comorbidity number	—	—	—	—	—	—
0	817 (15.28%)	922 (7.88%)	***<0.001***	472 (12.73%)	443 (11.94%)	0.580
1–2	2,181 (40.80%)	4,394 (37.55%)	—	1,525 (41.12%)	1,547 (41.71%)	—
≥3	2,348 (43.92%)	6,387 (54.58%)	—	1712 (46.16%)	1719 (46.35%)	—
Immunosuppressive therapy (IST)	—	—	0.397	—	—	0.589
No	5,159 (96.50%)	11,323 (96.75%)	—	3,569 (96.23%)	3,560 (95.98%)	—
Yes	187 (3.5%)	380 (3.25%)	—	140 (3.77%)	149 (4.02%)	—
Glucocorticoid usage	—	—	***0.027***	—	—	0.659
No	2,755 (51.53%)	5,817 (49.71%)		1892 (51.01%)	1911 (51.52%)	—
Yes	2,591 (48.47%)	5,886 (50.29%)		1817 (48.99%)	1798 (48.48%)	—
Haematopoietic growth factor therapy	—	—	***0.004***	—	—	0.674
No	3,948 (73.85%)	8,392 (71.71%)	—	2,718 (73.28%)	2,734 (73.71%)	—
Yes	1,398 (26.15%)	3,311 (28.29%)	—	991 (26.72%)	975 (26.29%)	—
Androgen therapy	—	—	0.611	—	—	0.759
No	5,279 (98.75%)	11,567 (98.84%)	—	3,659 (98.65%)	3,662 (98.73%)	—
Yes	67 (1.25%)	136 (1.16%)	—	50 (1.35%)	47 (1.27%)	—
Chemotherapy	—	—	***<0.001***	—	—	0.090
No	4,705 (88.01%)	10,506 (89.77%)	—	3,273 (88.24%)	3,319 (89.49%)	—
Yes	641 (11.99%)	1,197 (10.23%)	—	436 (11.76%)	390 (10.51%)	—
Iron chelation therapy	—	—	***0.016***	—	—	0.626
No	5,169 (96.69%)	11,393 (97.35%)	—	3,573 (96.33%)	3,565 (96.12%)	—
Yes	177 (3.31%)	310 (2.65%)	—	136 (3.67%)	144 (3.88%)	—
Blood transfusion therapy	—	—	***<0.001***	—	—	0.346
No	1879 (35.15%)	1,650 (14.1%)	—	1,078 (29.06%)	1,115 (30.06%)	—
Yes	3,467 (64.85%)	10,053 (85.9%)	—	2,631 (70.94%)	2,594 (69.94%)	—

*p*-value (*p* < 0.05) was highlighted in bold italic.

CHM, Chinese herbal medicine; N, number; CCI, Charlson comorbidity index; SD, standard deviation; IST, Immunosuppressive therapy; ATG, anti-thymocyte globulin; G-CSF, granulocyte colony-stimulated factor; ICD9-CM, the International Classification of Diseases, Ninth Revision, Clinical Modification.

Patients with aplastic anemia (ICD9-CM code: 284).

Immunosuppressive therapy (IST) included anti-thymocyte globulin (ATG) (ATC code: L04AA03 and L04AA04), cyclosporine (ATC code: L04AD01), eltrombopag (ATC code: B02BX05), alemtuzumab (ATC code: L04AA34), mycophenolate mofetil (ATC code: L04AA06), and sirolimus (L04AA10). IST drugs was used within 1 year before or after the diagnosed date of aplastic anemia.

Glucocorticoids included prednisolone (ATC code: H02AB06) and methylprednisolone (ATC code: H02AB04). Glucocorticoids were used within 1 year before or after the diagnosed date of aplastic anemia.

Haematopoietic growth factor therapy included granulocyte colony-stimulated factor (G-CSF) (ATC code: L03AA02, L03AA03, L03AA09, L03AA10, L03AA12, L03AA13, L03AA14, L03AA15, L03AA16, and L03AA17) and erythropoietin (EPO) (ATC code: B03XA01). Haematopoietic growth factor therapy were used within 1 year before or after the diagnosed date of aplastic anemia.

Androgen therapy included androgen (ATC code: G03B). Androgen therapy were used within 1 year before or after the diagnosed date of aplastic anemia.

Chemotherapy included cyclophosphamide (ATC code: L01AA01). Chemotherapy were used within 1 year before or after the diagnosed date of aplastic anemia.

Iron chelatiors (ATC code: V03AC01, V03AC02, and V03AC03). Iron chelatiors were used within 1 year before or after the diagnosed date of aplastic anemia.

Blood transfusion therapy included procedures (procedure code: 94001, 94005, 93001, 93002, 93003, 93019, 93004, 93007, 93016, and 93023C). Blood transfusion therapy were used within 1 year before or after the diagnosed date of aplastic anemia.

Propensity score matching method was performed for age, gender, Charlson comorbidity index score, immunosuppressive therapy, glucocorticoid usage, haematopoietic growth factor therapy, androgen therapy, chemotherapy, iron chelation therapy, and blood transfusion therapy (1:1 ratio).

### Chinese Herbal Medicine

Single herb and herbal formula are two types of CHM products used in AA patients. A single herb was a part of a plant, including flowers, fruits, seeds, stems, roots, and leaves. The organs of animals, insects, or minerals also can be used as the single herb. The herbal formula contained at least two single herbs. In our study, the CHM prescriptions (Table 2 and Supplementary Table S1) for AA patients were prescribed by licensed doctors of traditional Chinese medicine in Taiwan. These Chinese herbal medicine were manufactured by Taiwan’s pharmaceutical companies with the certificate of Good Manufacturing Practice (GMP) (Li et al., 2018; Cheng et al., 2019; Tsai et al., 2019).

**TABLE 2:** Composition of the most commonly used herbal formulas and single herbs for patients with aplastic anemia in Taiwan.

Formulas	Chinese name	Number of herbs	Composition (pin-yin name (latin name; botanical plant name))	Frequency of prescriptions	Person-year	Percentage of usage person	Avg. drug dose per day (g)	Average duration for prescription (days)
Total	—	—	—	121770	17492.00	100.00	13.40	8.84
Herbal formula (Pin-yin name)	—	—	—	116887	17438.20	99.27	9.53	8.78
Gui-Pi-Tang (GPT)	歸脾湯	12	**Ren-Shen** (*Radix Ginseng*; *Panax ginseng var. repens* (Maxim.) Makino), **Huang-Qi** (*Radix Astragali*; *Astragalus membranaceus* (Fisch.) Bunge), **Bai-Zhu** (*Rhizoma Atractylodis Macrocephalae*; *Atractylis macrocephala* (Koidz.) Hand.-Mazz.), **Fu-Ling** (*Poria*; *Wolfiporia cocos* (F.A. Wolf) Ryvarden & Gilb), **Suan-Zao-Ren** (*Semen Zizyphi Spinosae*; *Ziziphus jujuba f. lageniformis* (Nakai) Kitag.), **Long-Yan-Rou** (*Arillus Longan*; *Dimocarpus longan var. obtusus* (Pierre) Leenh.), **Mu-Xiang** (*Radix Aucklandiae*; *Himalaiella abnormis* (Lipsch.) Raab-Straube), **Gan-Cao** (*Radix Glycyrrhizae*; *Glycyrrhiza glabra var. glandulifera* (Waldst. and Kit.) Boiss.), **Dang-Gui** (*Radix Angelicae Sinensi*; *Angelica sinensis* (Oliv.) Diels), **Yuan-Zhi** (*Radix Polygalae*; *Polygala sibirica var. tenuifolia* (Willd.) Backer & Moore), **Sheng-Jiang** (*Rhizoma Zingiberis Recens*; *Zingiber officinale f. rubens* (Makino) M.Hiroe), **Da-Zao** (*Fructus Jujube*; *Ziziphus jujuba f. lageniformis* (Nakai) Kitag.)	8,588	6,442.00	31.52	4.78	10.87
**Single herbs (Pin-yin name)**	—	—	—	102198	17018.60	96.12	5.01	9.07
Dan-Shen (DanS)	丹參	1	**Dan-Shen** (*Radix Salviae Miltiorrhizae*; *Salvia miltiorrhiza var. charbonnelii* (H.Lév.) C.Y.Wu)	9,867	7,646.20	36.42	1.38	10.54
Huang-Qi (HQi)	黃耆	1	**Huang-Qi** (*Radix Astragali*; *Astragalus membranaceus* (Fisch.) Bunge)	9,058	7,262.00	34.65	1.52	10.45
Bai-Hua-She-She-Cao (BHSSC)	白花蛇舌草	1	**Bai-Hua-She-She-Cao** (*Herba Hedyotis Diffusae*; *Oldenlandia diffusa* (Willd.) Roxb.)	6,767	2,685.70	15.83	1.51	12.76
Ban-Zhi-Lian (BZL)	半枝蓮	1	**Ban-Zhi-Lian** (*Herba Scutellariae Barbatae*; *Scutellaria barbata* D. Don)	6,042	2,171.00	11.86	2.24	12.11
Xian-He-Cao (XHC)	仙鶴草	1	**Xian-He-Cao** (*Herba Agrimoniae*; *Agrimonia pilosa f. borealis* (Kitag.) Chu)	4,036	2,941.40	14.37	1.32	11.95
Dang-Gui (DG)	當歸	1	**Dang-Gui** (*Radix Angelicae Sinensi*; *Angelica sinensis* (Oliv.) Diels)	3,712	4,445.40	19.52	1.10	10.07

*Sorted by frequency of prescriptions.

Information are obtained from the websites (http://www.americandragon.com/index.htm; http://old.tcmwiki.com/; http://www.shen-nong.com/eng/front/index.html; http://www.ipni.org/; http://www.theplantlist.org/; http://www.worldfloraonline.org/).

### Association Rule

We used association rule to investigate the prescription profile of CHM (Yang et al., 2013). SAS software (version 9.4; SAS Institute, Cary, NC, United States) was used to implement the association rule as previous studies for CHM pairs (Wang CY. et al., 2020; Wu et al., 2020; Ho et al., 2021). The lift value, support value (X) (%), and confidence value (CHM_X→ CHM_Y; %) presented the association strength between CHM pairs (CHM products X and Y) as previous studies (Table 3).

**TABLE 3:** Most commonly used pairs of CHM products for patients with aplastic anemia in Taiwan.

CHM products (LHS, X)	Chinese name	Frequency of prescriptions of X product	—	CHM products (RHS, Y)	Chinese name	Frequency of prescriptions of Y product	Frequency of prescriptions of X and Y products	Support (X) (%)	Confidence (X →Y) (%)	Lift
Ban-Zhi-Lian (BZL; *Scutellaria barbata* D.Don)	半枝蓮	6,042	→	Ban-Zhi-Lian (BZL; *Scutellaria barbata* D.Don)	白花蛇舌草	6,767	3,661	3.01	60.59	10.90
Dang-Gui (DG; *Angelica sinensis* (Oliv.) Diels)	當歸	3,712	→	Huang-Qi (HQi; *Astragalus membranaceus* (Fisch.) Bunge)	黃耆	9,058	1778	1.46	47.90	6.44
Xian-He-Cao (XHC; *Agrimonia pilosa f. borealis* (Kitag.) Chu)	仙鶴草	4,036	→	Gui-Pi-Tang (GPT)	歸脾湯	8,588	1,345	1.10	33.33	4.73
Huang-Qi (HQi; *Astragalus membranaceus* (Fisch.) Bunge)	黃耆	9,058	→	Dan-Shen (DanS; *Salvia miltiorrhiza var. charbonnelii* (H.Lév.) C.Y.Wu)	丹參	9,867	1,241	1.02	13.70	1.69
Bai-Hua-She-She-Cao (BHSSC; *Scutellaria barbata* D.Don)	白花蛇舌草	6,767	→	Dan-Shen (DanS; *Salvia miltiorrhiza var. charbonnelii* (H.Lév.) C.Y.Wu)	丹參	9,867	1,207	0.99	17.84	2.20

CHM, Chinese herbal medicine; LHS, left-hand-side; RHS, right-hand-side.

Total prescriptions = 121770.

Support (X) (%) = Frequency of prescriptions of X and Y products/total prescriptions x 100%.

Confidence (X →Y) (%) = Frequency of prescriptions of X and Y products/Frequency of prescriptions of X product x 100%.

Lift = Confidence (X →Y) (%)/P (Y) (%).

P (Y) (%) = Frequency of prescriptions of Y product/total prescriptions x 100%.

Gui-Pi-Tang (GPT) was the herbal formula and was composed of 12 single herbs (Table 2).

Pin-yin and botanical plant names for Chinese herbs were obtained from the websites (http://www.americandragon.com/index.htm; http://old.tcmwiki.com/; http://www.shen-nong.com/eng/front/index.html; http://www.ipni.org/; http://www.theplantlist.org/; http://www.worldfloraonline.org/).

### Network Analysis

**FIGURE 3:**
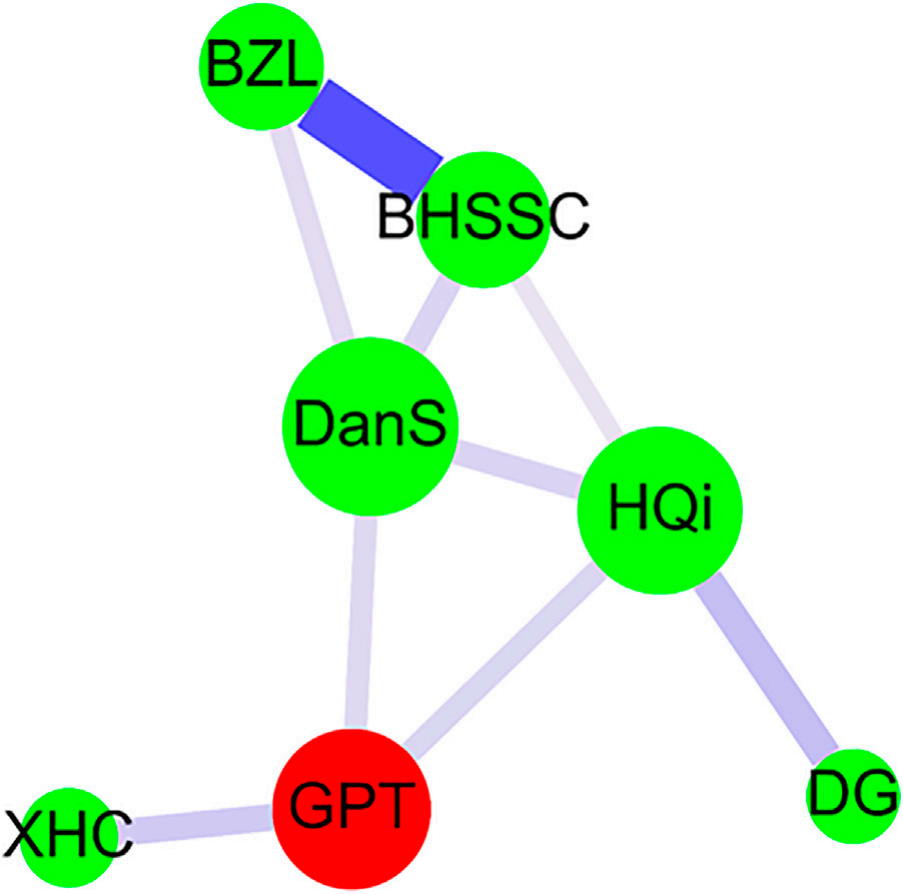
CHM network analysis in AA patients. Herbal formula is shown as the red circle, and single herb is expressed as the green circle. The circle size indicates prescription frequency of the CHM. The line size and color signify the support value and the lift value between paired CHM products, respectively. The thicker and darker connection line shows the stronger strength of connection between the paired CHM products. Abbreviations: AA, aplastic anemia; CHM, Chinese herbal medicine; BZL, Ban-Zhi-Lian (*Scutellaria barbata* D. Don); BHSSC, Bai-Hua-She-She-Cao [*Oldenlandia diffusa* (Willd.) Roxb.]; DanS, Dan-Shen [*Salvia miltiorrhiza var. charbonnelii* (H.Lév.) C.Y.Wu]; HQi, Huang-Qi [*Astragalus membranaceus* (Fisch.) Bunge]; DG, Dang-Gui [*Angelica sinensis* (Oliv.) Diels]; XHC, Xian-He-Cao [*Agrimonia pilosa f. borealis* (Kitag.) Chu]; GPT, Gui-Pi-Tang. Gui-Pi-Tang (GPT) was the herbal formula and was composed of 12 single herbs (Table 2). Pin-yin and botanical plant names for Chinese herbs were obtained from the websites (http://www.americandragon.com/index.htm; http://old.tcmwiki.com/; http://www.shen-nong.com/eng/front/index.html; http://www.ipni.org/; http://www.theplantlist.org/; http://www.worldfloraonline.org/).

Cytoscape was used to accomplish network analysis for CHM clusters as previous studies described (Yang et al., 2013; Wang CY. et al., 2020; Wu et al., 2020; Ho et al., 2021). A red circle indicated the herbal formula, and a green circle indicated a single herb. The prescription frequency of CHM was presented by the circle size. The support value between CHM pairs was signified by the line size. The lift value between CHM pairs was displayed by line color. The strength of stronger connection between CHM pairs was shown by the thicker and darker connection line (Figure 3).

### Statistical Analysis

Categorical data was shown as numbers (percentages), including age, gender, Charlson comorbidity number, immunosuppressive therapy (IST), glucocorticoid usage, hematopoietic growth factor therapy, androgen therapy, chemotherapy, iron chelation therapy, and blood transfusion therapy. The differences between CHM-users and non-users were evaluated by Chi-squared test (Table 1). Continuous data was shown as mean ± standard deviation, such as CCI score. The differences between CHM and non-CHM users were evaluated by Student’s t test (Table 1). The risks of overall and anemia-related mortalities in AA patients was estimated using the crude and adjusted Cox proportional hazard models (Table 4). We adjusted the confounding factors including age, gender, comorbidities, glucocorticoid usage, and the use of immunosuppressive therapy, hematopoietic growth factor therapy, androgen therapy, chemotherapy, iron chelation therapy, and blood transfusion therapy (Table 4). The cumulative incidence of overall mortality between CHM and non-CHM users was assessed by Kaplan‒Meier curves and log-rank tests (Supplementary Figure S1). *p*-values < 0.05 were considered statistically significant. SAS software (version 9.4; SAS Institute) was utilized to perform all of the analyses.

**TABLE 4:** Cox proportional hazard models for risk of overall and anemia-related mortalites in patients with aplastic anemia.

Characteristics	Risk of overall mortality							Risk of anemia-related mortality					
	Crude			Adjusted				Crude			Adjusted		
	HR	95% CI	*p*-value	aHR	95% CI	*p*-value		HR	95% CI	*p*-value	aHR	95% CI	*p*-value
Age (years old)	—	—	—	—	—	—	—		—	—	—	—	—
18≦Age<40 (vs. 0≦Age<18)	2.22	(1.75–2.81)	***<0.001***	2.54	(2.01–3.20)	***<0.001***	0.86		(0.37–1.98)	0.722	1.03	(0.44–2.41)	0.948
40≦Age<65 (vs. 0≦Age<18)	4.40	(3.58–5.41)	***<0.001***	4.11	(3.34–5.06)	***<0.001***	0.58		(0.28–1.18)	0.130	0.85	(0.40–1.82)	0.681
65≦Age (vs. 0≦Age<18)	6.26	(5.10–7.68)	***<0.001***	6.21	(5.02–7.68)	***<0.001***	1.80		(0.94–3.46)	0.079	3.47	(1.66–7.27)	***<0.001***
Female (vs. male)	0.77	(0.72–0.82)	***<0.001***	0.76	(0.71–0.81)	***<0.001***	0.69		(0.49–0.99)	***0.041***	0.73	(0.50–1.06)	0.102
CHM use (vs. non-CHM use)	0.74	(0.71–0.78)	***<0.001***	0.70	(0.66–0.74)	***<0.001***	0.49		(0.34–0.70)	***<0.001***	0.46	(0.32–0.67)	***<0.001***

CHM, Chinese herbal medicine; IST, immunosuppressive therapy; HR, hazard ratio; aHR, adjusted hazard ratio; 95% CI, 95% confidence interval.

Patients with aplastic anemia (ICD9-CM code: 284).

CHM use was adjusted by age, gender, comorbidities, and usages of immunosuppressive therapy, glucocorticoid usage, haematopoietic growth factor therapy, androgen therapy, chemotherapy, iron chelation therapy, and blood transfusion therapy.

Usages of therapies were applied within 1 year before or after the diagnosed date of aplastic anemia.

Significant *p*-values (*p* < 0.05) are highlighted in bold italic font.

## Results

### Demographic Characteristics

The demographic characteristics of patients with aplastic anemia (AA) in Taiwan were shown in Table 1. During the study period, there were 5,346 CHM users and 11,703 non-CHM users among AA patients in this study. Age, gender, comorbidities, glucocorticoid usage, and the use of hematopoietic growth factor therapy, chemotherapy, iron chelation therapy, and blood transfusion therapy were significantly different between CHM and non-CHM users (*p* < 0.05; Table 1). The propensity score was performed to decrease potential confounders. After matching, there were 3,709 CHM-users and 3,709 non-CHM users. There were no significant differences in demographic characteristics between these two groups after matching (*p* > 0.05; Table 1).

### Overall Mortality

The crude hazard ratios (HR) and adjusted hazard ratios (aHR) showed that the risk of overall mortality in AA patients significantly increased with age (Table 4). AA patients aged ≧65 years had 6.21 times higher overall mortality risk than those under 18 years of age even after adjusting for confounding factors [aHR: 6.21, 95% confidence interval (CI): 5.02-7.68, *p* < 0.001; Table 4]. Females presented a significantly lower overall mortality risk than males (HR: 0.77, 95% CI: 0.72-0.82, *p* < 0.001; aHR: 0.76, 95% CI: 0.71–0.81, *p* < 0.001; Table 4). The risk of overall mortality was significantly lower in CHM-users when compared to non-CHM users (aHR: 0.70, 95% CI: 0.66–0.74, *p* < 0.001; Table 4). The difference in the cumulative incidence of overall mortality between CHM and non-CHM users was displayed by Kaplan‒Meier survival plots (Supplementary Figure S1; *p* < 0.0001). CHM users presented a significantly lower cumulative incidence of overall mortality when compared to non-CHM users.

### Anemia-Related Mortality

AA patients over 65 years had 3.47 times higher anemia-related mortality risk when compared to those under 18 years of age after adjustment (aHR: 3.47, 95% CI: 1.66-7.27, *p* < 0.001; Table 4). Females presented a lower anemia-related mortality risk than males (HR: 0.69, 95% CI: 0.49-0.99, *p* = 0.041), but it is not significant after adjustment (Table 4). CHM-users presented a significantly lower anemia-related mortality risk when compared to non-CHM users (aHR: 0.46, 95% CI: 0.32-0.67, *p* < 0.001; Table 4).

### CHM Prescription Pattern

The prescription frequency and composition of CHM for AA patients in Taiwan was shown in Table 2. According to prescription frequency, Gui-Pi-Tang (GPT) was the most frequently used herbal formula. For single herbs, Dan-Shen [DanS; *Salvia miltiorrhiza var. charbonnelii* (H.Lév.) C.Y.Wu] was the most commonly used single herb, followed by Huang-Qi [HQi; *Astragalus membranaceus* (Fisch.) Bunge], Bai-Hua-She-She-Cao [BHSSC; *Oldenlandia diffusa* (Willd.) Roxb.], Ban-Zhi-Lian (BZL; *Scutellaria barbata* D. Don), Xian-He-Cao (XHC; *Agrimonia pilosa f. borealis* (Kitag.) Chu), and Dang-Gui (DG; *Angelica sinensis* (Oliv.) Diels).

To evaluate the effect of the efficacy of commonly prescribed herbs on overall and anemia-related mortalities, the crude and adjusted Cox proportional hazard models were performed in patients with AA (Table 5). For overall mortality, we observed that patients with CHM use had the statistical significance of a lower risk of overall mortality (aHR: 0.70, 95% CI: 0.66-0.74, *p* < 0.001; Table 5). Among these CHMs, patients with the use of Gui-Pi-Tang (GPT) had a lower risk of overall mortality when compared to non-GPT users (aHR: 0.63, 95% CI: 0.58-0.69, *p* < 0.001; Table 5). For single herbs, patients with the use of Dan-Shen (DanS; *Salvia miltiorrhiza var. charbonnelii* (H.Lév.) C.Y.Wu) had a lower risk of overall mortality when compared to non-DanS users (aHR: 0.58, 95% CI: 0.53-0.63, *p* < 0.001; Table 5). Patients with the use of Huang-Qi [HQi; *Astragalus membranaceus* (Fisch.) Bunge], Bai-Hua-She-She-Cao [BHSSC; *Oldenlandia diffusa* (Willd.) Roxb.], Ban-Zhi-Lian (BZL; *Scutellaria barbata* D. Don), Xian-He-Cao (XHC; *Agrimonia pilosa f. borealis* (Kitag.) Chu), and Dang-Gui (DG; *Angelica sinensis* (Oliv.) Diels) were found to have a lower risk of overall mortality when compared to the users of non-HQi (aHR: 0.57, 95% CI: 0.52-0.61, *p* < 0.001), non-BHSSC (aHR: 0.79, 95% CI: 0.71-0.88, *p* < 0.001), non-BZL (aHR: 0.79, 95% CI: 0.70-0.88, *p* < 0.001), non-XHC (aHR: 0.65, 95% CI: 0.58-0.73, *p* < 0.001), and non-DG (aHR: 0.50, 95% CI: 0.45-0.56, *p* < 0.001), respectively (Table 5). For anemia-related mortality, we observed that patients with CHM use had the statistical significance of a lower risk of anemia-related mortality (aHR: 0.46, 95% CI: 0.32–0.67, *p* < 0.001; Table 5). However, there were no significant protective effect against anemia-related mortality for these commonly prescribed herbs after adjusting for potential confounding factors (*p* > 0.05; Table 5).

**TABLE 5:** Cox proportional hazard models for risk of overall and anemia-related mortalities in patients with aplastic anemia (stratified by herb use).

	Risk of overall mortality						Risk of anemia-related mortality						
	Crude			Adjusted			Crude			Adjusted		
	HR	95% CI	*p*-value	aHR	95% CI	*p*-value	HR	95% CI	*p*-value	aHR	95% CI	*p*-value
CHM use	—	—	—	—	—	—	—	—	—	—	—	—
No	1.00	NA	NA	1.00	NA	NA	1.00	NA	NA	1.00	NA	NA
Yes	0.74	(0.71–0.78)	***<0.001***	0.70	(0.66–0.74)	***<0.001***	0.49	(0.34–0.70)	***<0.001***	0.46	(0.32–0.67)	***<0.001***
Gui-Pi-Tang (GPT)	—	—	—	—	—	—	—	—	—	—	—	—
No	1.00	NA	NA	1.00	NA	NA	1.00	NA	NA	1.00	NA	NA
Yes	0.62	(0.57–0.68)	***<0.001***	0.63	(0.58–0.69)	***<0.001***	1.14	(0.73–1.78)	0.555	0.82	(0.51–1.31)	0.395
Dan-Shen (DanS; *Salvia miltiorrhiza var. charbonnelii* (H.Lév.) C.Y.Wu)	—	—	—	—	—	—	—	—	—	—	—	—
No	1.00	NA	NA	1.00	NA	NA	1.00	NA	NA	1.00	NA	NA
Yes	0.57	(0.53–0.62)	***<0.001***	0.58	(0.53–0.63)	***<0.001***	0.76	(0.47–1.24)	0.276	0.81	(0.5–1.32)	0.402
Huang-Qi (HQi; *Astragalus membranaceus* (Fisch.) Bunge)	—	—	—	—	—	—	—	—	—	—	—	—
No	1.00	NA	NA	1.00	NA	NA	1.00	NA	NA	1.00	NA	NA
Yes	0.57	(0.52–0.61)	***<0.001***	0.57	(0.52–0.61)	***<0.001***	0.76	(0.47–1.25)	0.278	0.70	(0.42–1.16)	0.163
Bai-Hua-She-She-Cao (BHSSC; *Oldenlandia diffusa* (Willd.) Roxb.)	—	—	—	—	—	—	—	—	—	—	—	—
No	1.00	NA	NA	1.00	NA	NA	1.00	NA	NA	1.00	NA	NA
Yes	0.97	(0.87–1.08)	0.558	0.79	(0.71–0.88)	***<0.001***	0.37	(0.14–0.98)	***0.046***	0.42	(0.16–1.16)	0.094
Ban-Zhi-Lian (BZL; *Scutellaria barbata* D.Don)	—	—	—	—	—	—	—	—	—	—	—	—
No	1.00	NA	NA	1.00	NA	NA	1.00	NA	NA	1.00	NA	NA
Yes	0.97	(0.87–1.09)	0.608	0.79	(0.70–0.88)	***<0.001***	0.24	(0.06–0.98)	***0.046***	0.36	(0.09–1.49)	0.160
Xian-He-Cao (XHC; *Agrimonia pilosa f. borealis* (Kitag.) Chu)	—	—	—	—	—	—	—	—	—	—	—	—
No	1.00	NA	NA	1.00	NA	NA	1.00	NA	NA	1.00	NA	NA
Yes	0.65	(0.58–0.73)	***<0.001***	0.65	(0.58–0.73)	***<0.001***	1.19	(0.64–2.19)	0.580	0.90	(0.48–1.68)	0.739
Dang-Gui (DG; *Angelica sinensis* (Oliv.) Diels)	—	—	—	—	—	—	—	—	—	—	—	—
No	1.00	NA	NA	1.00	NA	NA	1.00	NA	NA	1.00	NA	NA
Yes	0.47	(0.42–0.53)	***<0.001***	0.50	(0.45–0.56)	***<0.001***	0.68	(0.35–1.35)	0.270	0.57	(0.28–1.15)	0.116

CHM, Chinese herbal medicine; HR, hazard ratio; aHR, adjusted hazard ratio; 95% CI, 95% confidence interval; NA, not applicable.

Patients with aplastic anemia (ICD9-CM code: 284).

Models adjusted for age, gender, comorbidities, and usages of immunosuppressive therapy, glucocorticoid usage, haematopoietic growth factor therapy, androgen therapy, chemotherapy, iron chelation therapy, and blood transfusion therapy.

Usages of therapies were applied within 1 year before or after the diagnosed date of aplastic anemia.

Significant *p*-values (*p* < 0.05) are highlighted in bold italic font.

Gui-Pi-Tang (GPT) was the herbal formula and was composed of 12 single herbs (Table 2).

Pin-yin and botanical plant names for Chinese herbs were obtained from the websites (http://www.americandragon.com/index.htm; http://old.tcmwiki.com/; http://www.shen-nong.com/eng/front/index.html; http://www.ipni.org/; http://www.theplantlist.org/; http://www.worldfloraonline.org/).

There were 3,709 patients who had 121,770 prescriptions provided by the doctors of traditional Chinese medicine in Taiwan (Table 3). The most frequently prescribed CHM pairs for AA patients in Taiwan was analyzed by using the association rule (Table 3). Higher levels of lift, confidence, and support values indicated stronger associations between CHM pairs. As shown, the most frequently prescribed CHM pairs were Ban-Zhi-Lian (BZL; *Scutellaria barbata* D. Don)→Bai-Hua-She-She-Cao (BHSSC; *Oldenlandia diffusa* (Willd.) Roxb.) (first co-prescription frequency: 3,661, support: 3.01%, confidence: 60.59%, lift: 10.90), followed by Dang-Gui (DG; *Angelica sinensis* (Oliv.) Diels)→Huang-Qi [HQi; *Astragalus membranaceus* (Fisch.) Bunge] (second co-prescription frequency: 1,778, support: 1.46%, confidence: 47.90%, lift: 6.44), and Xian-He-Cao (XHC; *Agrimonia pilosa f. borealis* (Kitag.) Chu)→Gui-Pi-Tang (GPT) (third co-prescription frequency: 1,345, support: 1.10%, confidence: 33.33%, lift: 4.73) (Table 3).

The prescription network of CHM for AA patients in Taiwan was presented by network analysis (Figure 3). Our results revealed that there was one main CHM cluster. In this one main cluster, Gui-Pi-Tang (GPT), Dan-Shen [DanS; *Salvia miltiorrhiza var. charbonnelii* (H.Lév.) C.Y.Wu], Huang-Qi (HQi; *Astragalus membranaceus* (Fisch.) Bunge), Dang-Gui [DG; *Angelica sinensis* (Oliv.) Diels], Bai-Hua-She-She-Cao [BHSSC; *Oldenlandia diffusa* (Willd.) Roxb.], Ban-Zhi-Lian (BZL; *Scutellaria barbata* D. Don), and Xian-He-Cao [XHC; *Agrimonia pilosa f. borealis* (Kitag.) Chu] were important CHMs for AA patients. Among them, the most frequently prescribed herbal formula and single herb were GPT and DanS, respectively (Table 2 and Figure 3). The strongest connection strength was between BHSSC and BZL, and the second one was between HQi and DG (Table 3 and Figure 3).

## Discussion

Chinese herbal medicines (CHMs) exhibit effective, safe, less toxic and few side-effects in bone marrow failure related diseases (Gao and Chong, 2012; Zhu N. et al., 2018; Sun et al., 2018; Liu WB. et al., 2019). Long-term CHM effect on aplastic anemia (AA) patients remains to be elucidated. In this study, we conducted a population-based retrospective cohort study to investigate the CHM effect on overall and anemia-related mortalities for these patients using a nationwide population-based database in Taiwan. We found that AA patients who used CHM had significantly lower risks of overall and anemia-related mortalities. Furthermore, we found that one CHM main cluster were important for these AA patient. This one main CHM cluster is composed by seven CHMs- Ban-Zhi-Lian (BZL; *Scutellaria barbata* D. Don), Bai-Hua-She-She-Cao [BHSSC; *Oldenlandia diffusa* (Willd.) Roxb.], Dan-Shen [DanS; *Salvia miltiorrhiza var. charbonnelii* (H.Lév.) C.Y.Wu], Huang-Qi[(HQi; *Astragalus membranaceus* (Fisch.) Bunge], Xian-He-Cao [XHC; *Agrimonia pilosa f. borealis* (Kitag.) Chu], Dang-Gui [DG; *Angelica sinensis* (Oliv.) Diels], and Gui-Pi-Tang (GPT). These results may suggest that CHMs exhibit the protective effect against overall and anemia-related mortalities in AA patients and may provide the utility of clinical CHM as an alternative therapy for the possible combination with the current conventional therapies for these patients.

We found that AA patients with over 65 years old had higher overall and anemia-related mortalities in Taiwan. For overall mortality, AA patients over 65 years old had a higher risk of 6.21-fold than those aged younger than 18 years old. This is consistent with previous studies (Vaht et al., 2017; Contejean et al., 2019). Contejean et al., reported that age is independently associated with mortality (Contejean et al., 2019). Vaht et al., reported that AA patients with over 60 years old have a poor 5-years survival rate of 38.1% (Vaht et al., 2017). Moreover, we firstly found the risk of anemia-related mortality in AA patients over 65 years old was 3.47-fold higher than those aged under 18 years old. No similar studies have been reported in anemia-related mortality in AA patients. Our results suggest that age is an independent risk factor for both of the overall and anemia-related mortalities in AA patients, especially for those who older than 60 years of age.

We also found that among AA patients, patients who used CHMs had lower risks of overall and anemia-related mortalities even after adjusting for age, gender, comorbidities, and usages of immunosuppressive therapy, glucocorticoid usage, hematopoietic growth factor therapy, androgen therapy, chemotherapy, iron chelation therapy, and blood transfusion therapy. CHMs may show the protective effect against bone marrow failure related diseases (Gao and Chong, 2012; Kuang et al., 2016; Sun et al., 2018; Jiang et al., 2021). Ginseng extract and its active component-panaxadiol saponins promote the proliferation and differentiation of hematopoietic progenitor cells and then regulate the immune function (Gao and Chong, 2012; Kuang et al., 2016; Sun et al., 2018; Jiang et al., 2021).

For AA patients in Taiwan, we found one main CHM cluster composed by seven CHMs- BZL (*Scutellaria barbata* D. Don), BHSSC [*Oldenlandia diffusa* (Willd.) Roxb.], DanS [*Salvia miltiorrhiza var. charbonnelii* (H.Lév.) C.Y.Wu], HQi [*Astragalus membranaceus* (Fisch.) Bunge], XHC [*Agrimonia pilosa f. borealis* (Kitag.) Chu], DG [*Angelica sinensis* (Oliv.) Diels], and GPT. The strongest connection strength was between BZL and BHSSC. Ban-Zhi-Lian (BZL), also named *Scutellaria barbata* D. Don, is a flowering plant of family Lamiaceae. Polysaccharides from BZL exhibit anti-complement activity and have been recommended to treat complement-associated diseases (Wu and Chen, 2009; Wu et al., 2009). Bai Hua She She Cao (BHSSC), also known as *Oldenlandia diffusa* (Willd.) Roxb., belongs to family Rubiaceae (Liang et al., 2008). BHSSC promotes blood circulation, clears heat away, removes dampness, and eliminates toxins. BHSSC also has immuno-modulating, anti-inflammatory, and anti-cancer effects (Shan et al., 2001; Gupta et al., 2004; Zhu et al., 2018a). For single herbs, DanS [*Salvia miltiorrhiza var. charbonnelii* (H.Lév.) C.Y.Wu] was the most prescribed single herb in our study. DanS is the dry root and rhizome of *Salvia miltiorrhiza var. charbonnelii* (H.Lév.) C.Y.Wu of family Labiatae (Leung et al., 2010). DanS promotes blood flow and circulation, widens blood vessels, and prevents platelet and blood clotting. Tanshinones are isolated from DanS, regulate metabolic function, and provide vasodilation, neuroprotection, anti-oxidation, anti-inflammation, anti-tumor, and phytoestrogenic activities (Wang X. et al., 2020). Also from DanS, salvianolic acid B shows anti-oxidant, anti-inflammatory, and anti-cancer activities (Shi et al., 2019).

The second most commonly used CHM pairs were HQi and DG. The combination of DG and HQi is widely used to treat iron-deficiency anemia, because it could increase iron through the biosynthesis of ferritin and improve the level of hemoglobin (Huang et al., 2016). Furthermore, DG and HQi recover the function of hematopoietic stem cells, balance T lymphocytes, inhibit the apoptosis of bone marrow cells induced by immune attack, and restore the balance of the T cell immune response (Liu J. et al., 2019). HQi, also known as *Astragalus membranaceus* (Fisch.) Bunge, is a flowering plant in the family Fabaceae. HQi is effective to treat chronic aplastic anemia by promoting the recovery of haematopoietic function through improving T-lymphocyte subsets and reducing the release of negative regulatory factors such as tumor necrosis factor-alpha (TNF-alpha) and interleukin-2 (IL-2) (Wang et al., 2007). Animal experiment shows that HQi increases serum megakaryocyte colony-stimulating activity (Meg-CSA) of anemic mice and accelerates the recovery of megakaryocyte hematopoiesis (Zhu and Zhu, 2001). Meta-analysis also reports that HQi significantly enhances the efficacy of androgens for aplastic anemia without severe side effects (Zhu et al., 2015). Dang-Gui (DG), also known as *Angelica sinensis* (Oliv.) Diels, belongs to the family Apiaceae. Angelica sinensis polysaccharide (ASP) from DG, is effective for the treatment of aplastic anemia (Chen et al., 2020). ASP could prevent mitochondrial apoptosis to restore the function of hematopoietic stem cells by suppressing abnormal T-cell immunity. Moreover, ASP inhibits NF-κB p65 activation via the IκB kinases- (IKKs-) IκBα pathway, thereby reducing the secretion of interleukin-6 (IL-6) and TNF-α, which is known to inhibit erythropoiesis (Wang et al., 2017).

The third most commonly used CHM pairs were between GPT and XHC. Gui-Pi-Tang (GPT) contains 12 single herbs. We found that GPT was the most commonly used CHM herbal formula for AA patients. Similar studies also reports that GPT is the most commonly prescribed CHM formula for treating anemia, acute myeloid leukemia, and osteopenia via hematopoiesis-stimulating activities (Kanai et al., 2005; Fleischer et al., 2017; Chen et al., 2018). It is also clinically prescribed to treat chronic immune thrombocytopenic purpura via autoantibodies suppression activities (Yamaguchi et al., 1993). XHC, also known as *Agrimonia pilosa f. borealis* (Kitag.) Chu, is a flowering plant of family Rosaceae. XHC exhibits anti-inflammatory, anti-oxidant and antimicrobial activities (Kim et al., 2017; Kim et al., 2020).

This study demonstrated that complementary CHM therapy may reduce overall and anemia-related mortalities among patients with AA. There are seven clinically used CHM products that are potentially useful for AA patients. However, the actual dose of specific CHMs in this prescription for patients was unknown, and the metabolism of the co-prescription pattern in humans, as well as potential confounders (i.e., body mass index, fatty tissue, lifestyles, personalized treatments, and social-economic status etc.) are not clarified in this study. Therefore, further randomized controlled trials and functional investigations of these potentially useful CHM products are necessary to validate their efficacy and safety in these patients.

## Data Availability

The datasets presented in this article are not readily available because Only a limited number of databases allowed access to raw data from the Taiwanese NHIRD database. Requests to access the datasets should be directed to W-ML, wmliang@mail.cmu.edu.tw.
