# Supplementary material for: Chinese Herbal Medicine Therapy Reduces the Risks of Overall and Anemia-Related Mortalities in Patients With Aplastic Anemia: A Nationwide Retrospective Study in Taiwan
Source: Front Pharmacol. 2021 Oct 8;12:730776. doi: 10.3389/fphar.2021.730776 (PMC8531749; doi:10.3389/fphar.2021.730776)
Supplement: Supplementary file 1 [file DataSheet1.docx]

**Supplementary Information**

Supplementary Text

Figure S1

Table S1

**Supplementary Text**

**Figure S1.** Kaplan‒Meier curves for overall mortality for CHM-users and non-users among AA patients. Abbreviations: CHM: Chinese herbal medicine; AA, aplastic anemia.

**Table S1**. Therapeutic actions and indications for the most commonly used herbal formulas and single herbs for patients with aplastic anemia in Taiwan.

**Figure S1.**

**
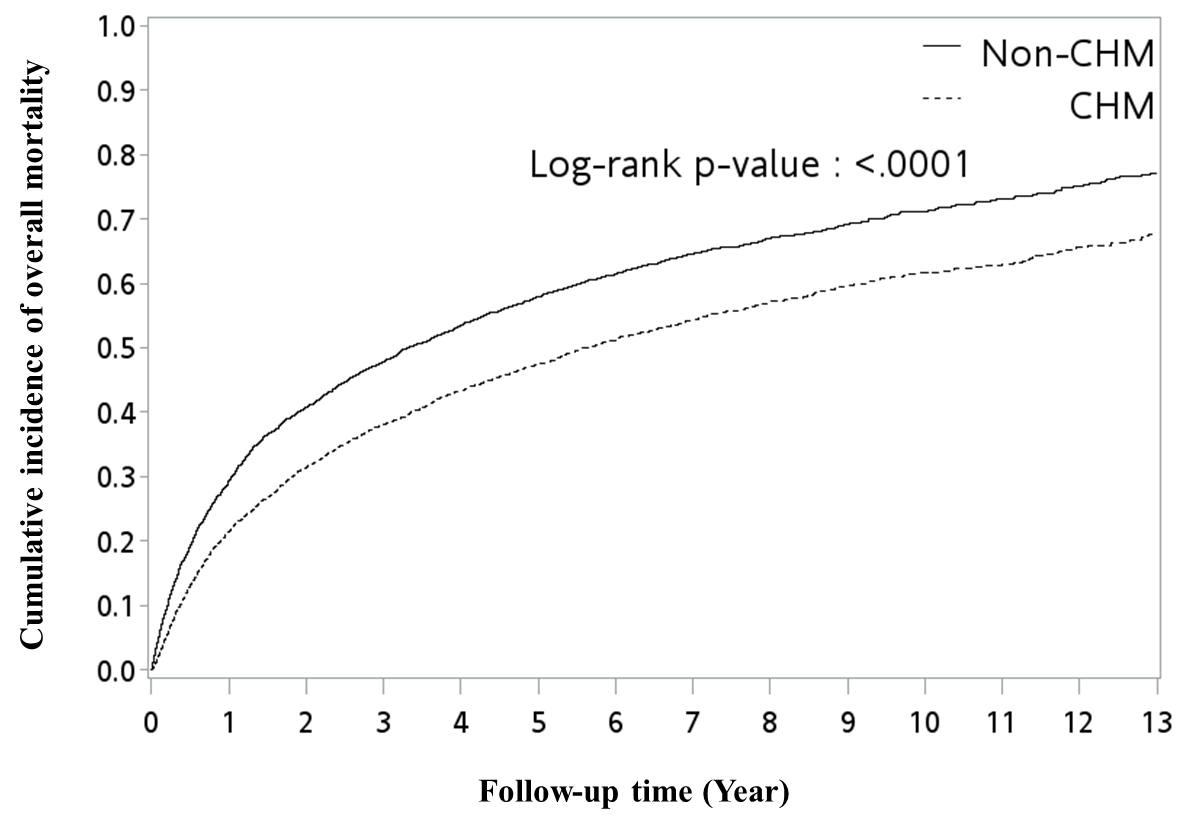
**

| **TABLE S1 \| Therapeutic actions and indications for the most commonly used herbal formulas and single herbs for patients with aplastic anemia in Taiwan** | | | | |
| --- | --- | --- | --- | --- |
| **Formulas** | **Chinese name** | **Number of herbs** | **Composition (Pin-yin name (latin name; botanical plant name))** | **Therapeutic actions and indications** |
| **Total** |  |  |  |  |
| **Herbal formula (Pin-yin name)** |  |  |  |  |
| Gui-Pi-Tang (GPT) | 歸脾湯 | 12 | **Ren-Shen** (*Radix Ginseng*; *Panax ginseng var. repens* (Maxim.) Makino), **Huang-Qi** (*Radix Astragali*; *Astragalus membranaceus* (Fisch.) Bunge), **Bai-Zhu** (*Rhizoma Atractylodis Macrocephalae*; *Atractylis macrocephala* (Koidz.) Hand.-Mazz.), **Fu-Ling** (*Poria*; *Wolfiporia cocos* (F.A. Wolf) Ryvarden & Gilb), **Suan-Zao-Ren** (*Semen Zizyphi Spinosae*; *Ziziphus jujuba f. lageniformis* (Nakai) Kitag.), **Long-Yan-Rou** (*Arillus Longan*; *Dimocarpus longan var. obtusus* (Pierre) Leenh.), **Mu-Xiang** (*Radix Aucklandiae*; *Himalaiella abnormis* (Lipsch.) Raab-Straube), **Gan-Cao** (*Radix Glycyrrhizae*; *Glycyrrhiza glabra var. glandulifera* (Waldst. & Kit.) Boiss.), **Dang-Gui** (*Radix Angelicae Sinensi*; *Angelica sinensis* (Oliv.) Diels), **Yuan-Zhi** (*Radix Polygalae*; *Polygala sibirica var. tenuifolia* (Willd.) Backer & Moore), **Sheng-Jiang** (*Rhizoma Zingiberis Recens*; *Zingiber officinale f. rubens* (Makino) M.Hiroe), **Da-Zao** (*Fructus Jujube*; *Ziziphus jujuba f. lageniformis* (Nakai) Kitag.) | Immuno-modulating and hematopoiesis-stimulating activities (Kanai et al., 2005; Fleischer et al., 2017; Chen et al., 2018; Yamaguchi et al., 1993) |
| **Single herbs (Pin-yin name)** |  |  |  |  |
| Dan-Shen (DanS) | 丹參 | 1 | **Dan-Shen** (*Radix Salviae Miltiorrhizae*; *Salvia miltiorrhiza var. charbonnelii* (H.Lév.) C.Y.Wu) | Anti-oxidant, anti-inflammatory, and anti-cancer activities (Shi et al., 2019; Wang et al., 2020b) |
| Huang-Qi (HQi) | 黃耆 | 1 | **Huang-Qi** (*Radix Astragali* ; *Astragalus membranaceus* (Fisch.) Bunge) | Immuno-modulating and hematopoiesis-stimulating activities (Wang et al., 2007; Zhu and Zhu, 2001) |
| Bai-Hua-She-She-Cao (BHSSC) | 白花蛇舌草 | 1 | **Bai-Hua-She-She-Cao** (*Herba Hedyotis Diffusae*; *Oldenlandia diffusa* (Willd.) Roxb.) | Immuno-modulating, anti-inflammatory, and anti-cancer activities (Shan et al., 2001; Gupta et al., 2004; Zhu et al., 2018a) |
| Ban-Zhi-Lian (BZL) | 半枝蓮 | 1 | **Ban-Zhi-Lian** (*Herba Scutellariae Barbatae*; *Scutellaria barbata* D. Don) | Anti-complement activity (Wu and Chen, 2009; Wu et al., 2009) |
| Xian-He-Cao (XHC) | 仙鶴草 | 1 | **Xian-He-Cao** (*Herba Agrimoniae*; *Agrimonia pilosa f. borealis* (Kitag.) Chu) | Anti-inflammatory, anti-oxidant and antimicrobial activities (Kim et al., 2017; Kim et al., 2020) |
| Dang-Gui (DG) | 當歸 | 1 | **Dang-Gui** (*Radix Angelicae Sinensi*; *Angelica sinensis* (Oliv.) Diels) | Immuno-modulating and anti-inflammatory activities (Chen et al., 2020; Wang et al., 2017) |
| *Sorted by frequency of prescriptions. | | | | |
| Information are obtained from the websites (http://www.americandragon.com/index.htm; http://old.tcmwiki.com/; http://www.shen-nong.com/eng/front/index.html; http://www.ipni.org/; http://www.theplantlist.org/). | | | | |
